# The discovery of the church of Rungholt, a landmark for the drowned medieval landscapes of the Wadden Sea World Heritage

**DOI:** 10.1038/s41598-024-66245-0

**Published:** 2024-07-06

**Authors:** Dennis Wilken, Hanna Hadler, Bente Sven Majchczack, Ruth Blankenfeldt, Oliver Auge, Sarah Bäumler, Dirk Bienen-Scholt, Ulf Ickerodt, Stefanie Klooß, Antonia Reiß, Timo Willershäuser, Wolfgang Rabbel, Andreas Vött

**Affiliations:** 1 Institute of Geosciences, Kiel University, Kiel, Germany; 2 Institute for Geography, Johannes Gutenberg-University Mainz, Mainz, Germany; 3 Cluster of Excellence ROOTS, Kiel University, Kiel, Germany; 4 Leibniz-Zentrum für Archäologie (LEIZA), Mainz, Germany; 5 Centre for Baltic and Scandinavian Archaeology (ZBSA), Schleswig, Germany; 6 Department of Regional History, Kiel University, Kiel, Germany; 7 State Archaeology Department Schleswig-Holstein, Schleswig, Germany

**Keywords:** Environmental social sciences, Environmental impact

## Abstract

The UNESCO World Heritage *Wadden Sea* holds remains of a medieval cultural landscape shaped by interactions between man and natural forces. From the Netherlands to Denmark, human efforts of cultivating low-lying areas created a unique coastal landscape. Since the Middle Ages, storm floods widely drowned embanked cultural land and especially affected North Frisia (Germany), where once fertile marshland was permanently turned into tidal flats. One key region, the Edomsharde, was widely destroyed in 1362 AD. Medieval settlement remains still occur in the tidal flats around the island Hallig Südfall and are commonly associated with Edomsharde’s trading centre Rungholt—ever since a symbol for the region’s drowned landscapes and focus of this study. We present a first-time comprehensive reconstruction of this medieval settlement by means of new geophysical, geoarchaeological and archaeological data. Our results reveal remains of up to 64 newly found and rectified dwelling mounds, abundant drainage ditches, a seadike, and especially the discovery of Edomshardes’s main church as important landmark in this former cultural landscape. These finds together with the documented imported goods confirm a thriving society, involved in transregional trade and thereby close a significant gap in medieval history not only for North Frisia, but the entire Wadden Sea region.

## Introduction

The UNESCO World Heritage Site *Wadden Sea* extends from the Netherlands to southern Denmark and is a globally unique ecosystem in a highly dynamic landscape^1^. During the Holocene, a natural dynamic system of tidal flats, adjacent salt marshes and fenlands formed an extensive amphibious transition zone between the open North Sea and the Pleistocene mainland^2^. In contrast, the Wadden Sea region’s present appearance is also the relic of a cultural landscape intensively shaped by the interaction between man and natural forces^3,4^.

Intense human efforts to reclaim the low-lying coastal areas comprised complex measures like diking, drainage and peat extraction, all together creating a man-made cultural landscape with similar patterns along the North Sea coast^3,5^. But cultivation finally culminated in a series of self-enhancing processes, eventually rendering this landscape highly vulnerable to flooding^5,6^: The indispensable artificial drainage of embanked marsh- and fenlands induced compaction, subsidence, and thus lowering of the ground surface^7,8^, as did the extraction of peat for melioration and heating^9,10^. In addition, the restriction of the North Sea’s flooding space by dikes caused an increased tidal range and higher storm surge levels^5^. As a result, storm floods since the Late Middle Ages repeatedly hit the artificially lowered coastal areas causing major losses of embanked cultural land.

**Figure 1:**
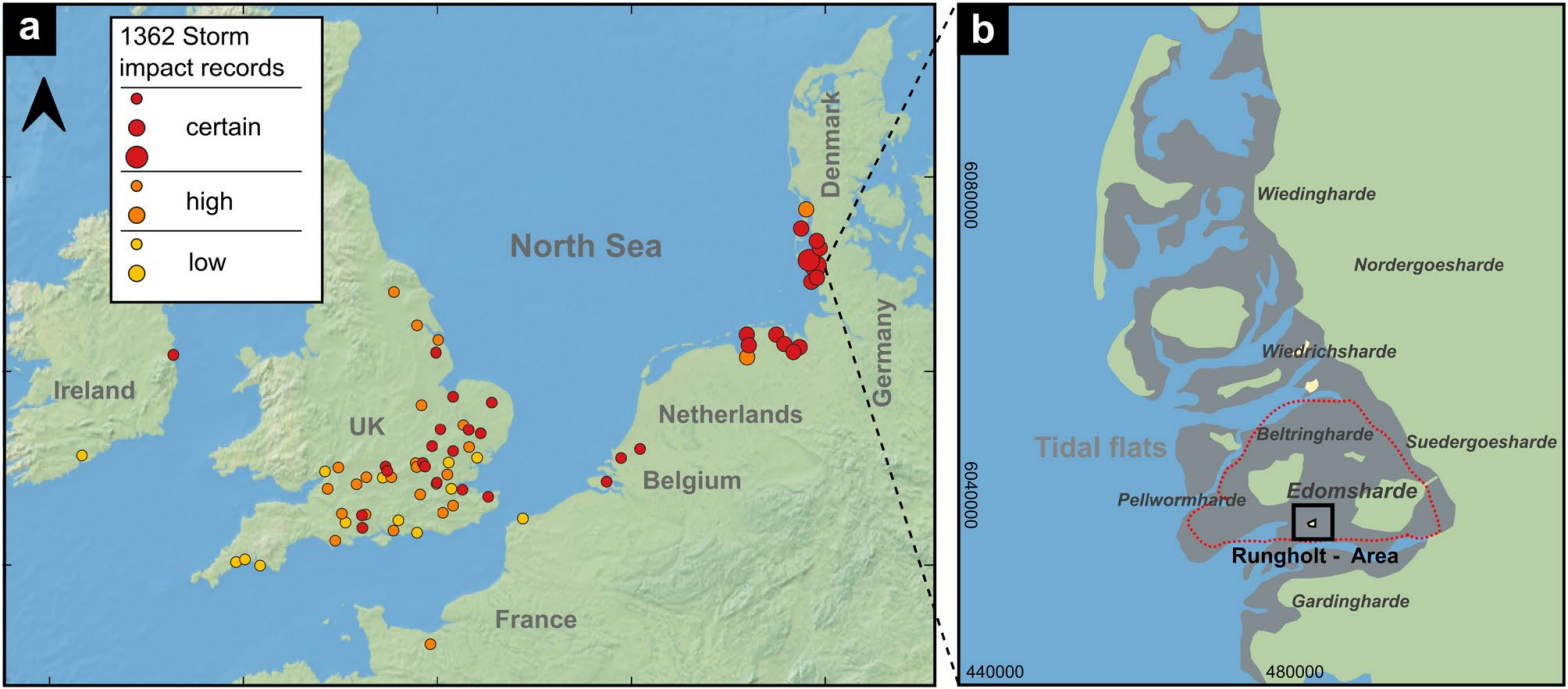
(**a**) Map of the North Sea region with sites impacted by the 1362 AD extreme storm and flood event (‘Grote Mandränke’) indicated by colored dots based on historical sources^12–16,73^. Dot colours reflect a confidence rating of the data and dot size represents the event’s impact with respect to landscape changes (small: short term flooding, damaged buildings; large: loss of land). The lack of records on the Belgian-Dutch coast is striking and may be caused by a lack of serious damage as a result of the storm’s track. (Background map made with Natural Earth. Free vector and raster map data naturalearthdata.com). (**b**) Map of North Frisia with approximate positions of the former Harden^36^. Grey areas show the tidal flats and green areas show the recent main-land^23^. The red dotted line indicates the presumably once diked area (after historical maps^36^) around the investigation area (marked with a black rectangle). Coordinates are in UTM32N (EPSG:32632).

Amongst the well known storm floods is the so-called 1st ‘Grote Mandränke’ (or 2nd Marcellus’ flood) that presumably occurred on January 16, 1362 AD^11^ (Fig. 1a). Though reliably documented for only some regions^12–15^, this storm like its many successors ravaged most of the North Sea region from SW to NE^16^. In Wadden Sea regions like Flanders^17^, Northern Holland^18^, Friesland^7^, Denmark^19^, and Britain^16^ medieval storms caused major coastal damage. While several of the impacted areas could be retrieved, large parts of East Frisia^12^ and especially North Frisia^20–23^ could not be reclaimed but irreversibly turned from fertile marshland to tidal flats.

In North Frisia, some coastal lowlands were already reclaimed by Frisian immigrants as early as the 8th cent. AD^24^. But the systematic large-scale reclamation and major transformation of natural coastal conditions into a cultural landscape had only begun around 1100 AD. Unlike in neighbouring Wadden Sea regions, prevailing amphibious conditions had restricted permanent human settlement for a long time^22^. Pleistocene outcrops and Holocene beach ridges had mostly kept out open North Sea conditions during the post-glacial sea level rise^2,25^. Instead, quiescent shallow water conditions and wide marshes emerged since c. 2000 BC, traversed by tidal channels and succeeded by extensive peat formation that lasted at least until the first millennium AD^22,25,26^. Yet, only two centuries of intense human interventions by medieval colonization increased the region’s vulnerability in a way that the devastating effects of the 1362 AD storm flood destroyed much of North Frisia’s low-lying coastal areas^11,21,27^. Recent (geo-)archaeological case studies on North Frisia’s landscape development imply that it is in particular large-scale peat extraction—for cultivation of fossil marshes in southern North Frisia (Südfall area)^22,27^ and salt production in the northern Halligen region^28^—that significantly lowered the ground surface, likely into the range of mean high water at that time.

The 1362 AD storm flood not only caused a hiatus in North Frisia’s stratigraphy but also in historical records. Hence, reliable written sources for this period are scarce , whereas numerous archaeological finds like import goods^29,30^, rare metal objects^30^, and massive coastal infrastructure^21,23^ indicate a thriving society involved in transregional maritime trade. Nevertheless, our knowledge about the size and extend of the medieval settlements and their (supra-)regional connections remains vague. The Danish kingdom appears to be the driving force for the medieval colonization and solicitation of Frisian settlers^31^. Besides the establishment of new settlements, cultural landscape development goes along with parish organization and establishment of new churches. This ecclesiastical development was obviously accompanied by the establishment and expansion of the secular administration by setting up so-called ‘*harden*’ as royal administrative districts in North Frisia^32^. From them, the Danish king received more income than from other parts of his domain^33,34^. The region’s great economic success was likely based on animal husbandry, farming and salt production from peat. Close connections with an international trading network are implied by lively maritime trade with German Hanseatic cities and also Flanders as, e.g., documented for the *Edomsharde*^33^.

It is also the Edomsharde, that was apparently strongly affected by land losses and widely destroyed in 1362 AD^35,36^. For the ‘Strand’ provostry alone, which included the Edomsharde, a 1436/37 AD income register of the Schleswig diocese lists the permanent flooding of 24 churches and chapels since 1362 AD. In this list, the parish of Rungholt stands out as the only one with a main church and so-called ‘*collegium*’, interpreted as regional ecclesiastical assembly^37,38^ or even collegiate church^34,39^. Rungholt’s function as the clerical but also judicial and administrative centre of the Edomsharde are historically documented at least for the 14th cent. AD^33,38^. Its apparent importance likely favoured the historically grown legend, placing Rungholt not only in the center of the 1362 AD disaster but at the same time making it a prominent example for the devastating effects of human intervention in coastal areas^40^.

In today’s tidal flats, remnants of the drowned medieval landscape are still preserved underneath recent sediments. Occasionally exposed remains around the marsh island Hallig Südfall (Fig. 1b, in the center of the black box labelled ‘Rungholt area’) were documented and mapped since the early 20th century^41^ and are commonly associated with Edomsharde’s medieval trading centre Rungholt. They include remains of dwelling mounds (so-called *terps*), abundant drainage ditches and even a dike with tidal gates^21,42^.

The archaeological potential and value of North Frisia’s Wadden Sea have been recognised since the 1970s. Though archaeological remains commonly suffer from (strong) erosion^23,43^, Holocene coastal dynamics were here less intense than, for example, in East Frisia. There, fossil surfaces became widely reworked by natural processes^44^. Although preconditions in North Frisia favoured the conservation of detailed (geo-)archaeological information on the cultural landscape, the tidal flat’s sheer size and prevailing natural conditions (e.g., tides, weather, accessibility) prevented a systematic and large-scale archaeological investigation. Despite their unique preservation potential and decades of archaeological work, so far neither a comprehensive reconstruction of the medieval settlement associated with Rungholt nor the rediscovery of the Edomshardes’s main church as an important landmark in this former cultural landscape have been successful.

To overcome the non-systematic surveying and close a major gap in medieval history not only for North Frisia, but the entire Wadden Sea region called for a novel interdisciplinary approach.

By combining state-of-the-art geophysical, geoarchaeological and archaeological methods^22,23,27^, we aimed at:

- The systematic, large-scale multi-method survey and identification of medieval archaeological cultural remains preserved in the tidal flats. The basis of our prospection work is multi-channel magnetic gradiometry, which is applied during low tide. Starting from a coarse search grid of 20 m profile distance to locate possible settlement remains like e.g. dwelling mounts, these remains are then imaged thoroughly by a comprehensive measurement grid. At key locations, the magnetic map is complemented by Electromagnetic Induction (EMI) measurements^45^ or marine seismic reflection profiles^23^. For details, see the “Methods” section below.
- The reconstruction of the settlement area associated with Rungholt and its (cultural) landscape development by means of geoarchaeological methods, namely vibracoring and subsequent state of the art palaeoenvironmental parameter analysis in combination with geophysical data inversions.
- A critical evaluation of the actual importance of this settlement in the light of the recent church discovery, and assessing the settlement’s role and importance in the center of a well known and historically attested wealthy medieval coastal region.

The Rungholt area thereby acts as a model region for the investigation and reconstruction of drowned medieval landscapes in the whole Wadden Sea region.

## Results

### Bringing back a drowned settlement

**Figure 2:**
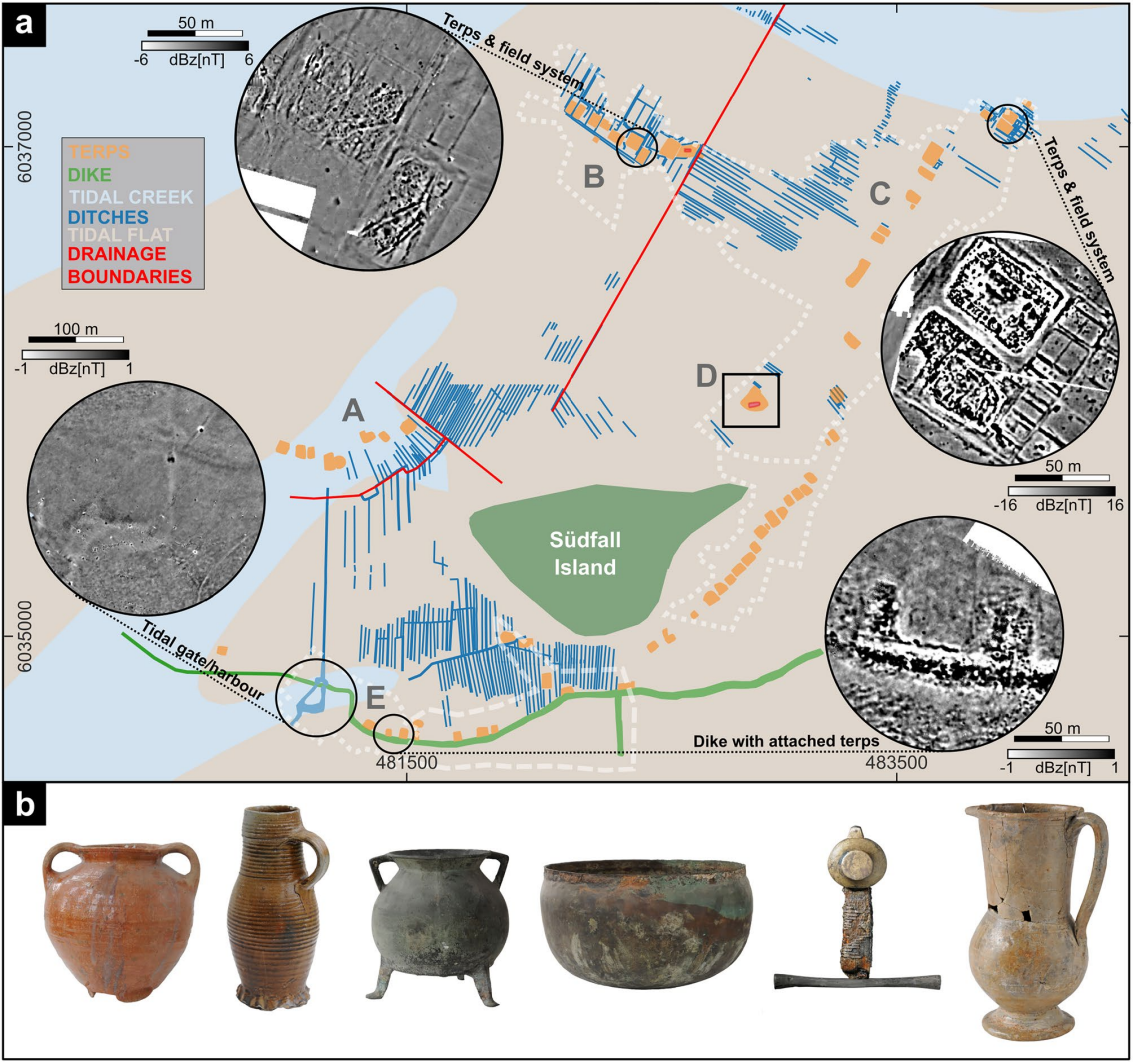
(**a**) The Rungholt settlement and investigation area. White dotted lines indicate the areas covered by magnetic gradiometry, the white dashed line outlines an area covered by marine seismic reflection profiles^23^. The insets highlight exemplary settlement structures as found by magnetic gradiometry for areas B (two terps and surrounding drainage ditches), C (two terps and ditch system), and E (dike section with attached terps^23^). Area A is flooded today but was archaeologically documented already in 1923^41^ and newly rectified in the present GIS project using QGIS 3.34.4 (https://www.qgis.org/en/site/). Red lines indicate boundaries of drainage direction change. Coordinates are in UTM32N (EPSG:32632). (**b**) Most common find categories of imported and high-quality objects have been found evenly distributed throughout the areas A, B and E. They include (from left to right, not to scale) imported lead-glazed redware and stoneware^30^, cast bronze cauldrons^30^, hammered brass cauldrons^30^, swords and hispano-moresque faience^30^ (the last only in area B). Areas C and D yield no finds due to sediment cover.

**Figure 3:**
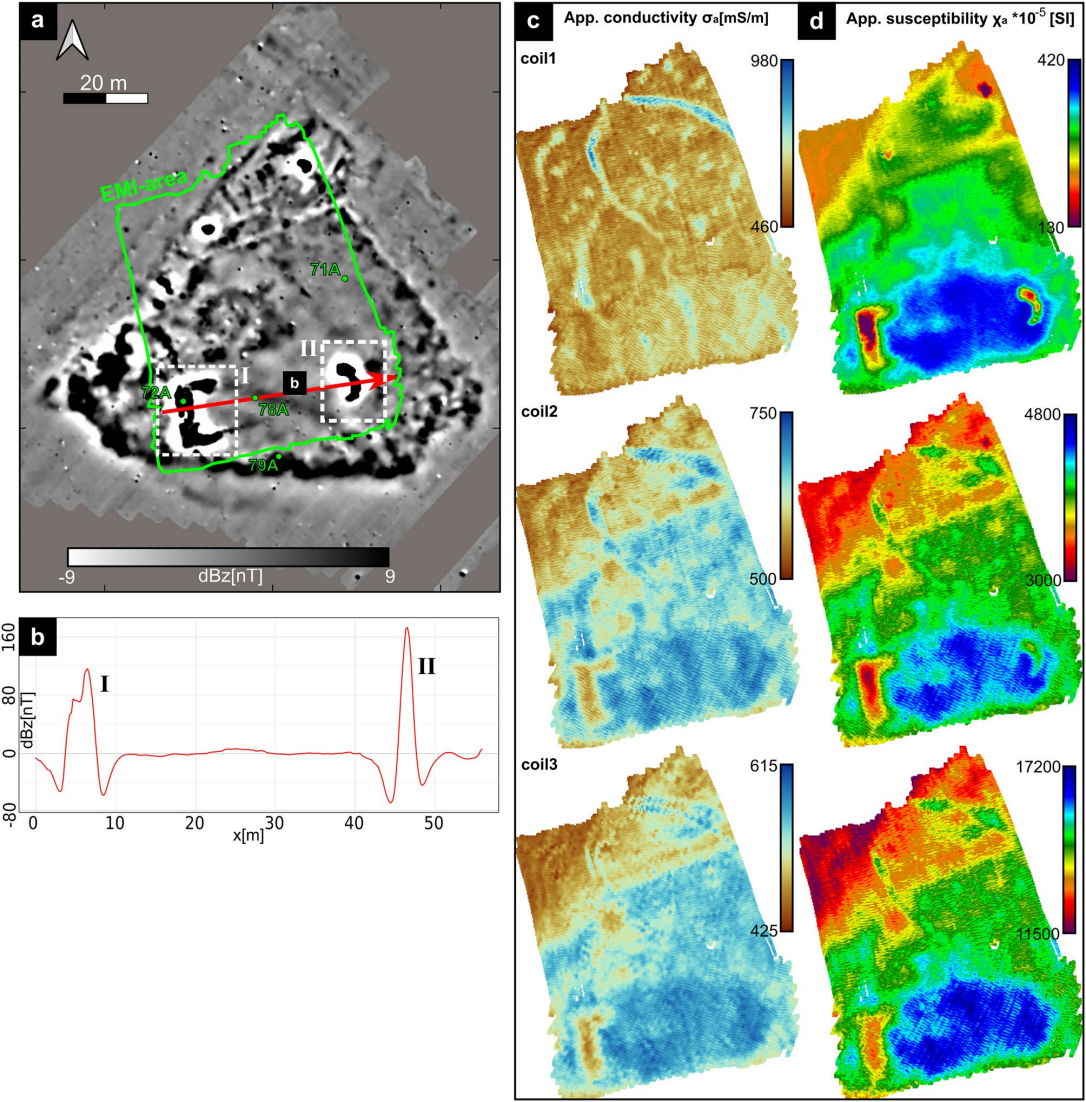
(**a**) Magnetic gradiometry map of site D (for location see Fig. 2). Green dots show coring sites. Red arrow indicates example profile seen in (**b**) highlighting the amplitude of the largest observed magnetic anomalies (labelled I and II) . (**c**) Maps of apparent electric conductivity for all three EMI coil distances, as well as (**d**) corresponding maps of apparent magnetic susceptibility (calibrated).

In the Rungholt region, our approach of joint geophysical, geoarchaeological and archaeologial prospection revealed numerous so far unknown medieval cultural remains. Using our new results and GIS-based analyses of formerly documented remains^30,41^, for the first-time allows us to interconnect isolated settlement structures to a large scale reconstruction of a medieval settlement area that extends over at least 10 km$^2$ (Fig. 2a). This reconstruction now reveals four different areas of settlement (A, B, C, and E in Fig. 2a), each a village with several rectangular terps arranged in a row. They are fitted into a characteristic drainage network of long ditches, forming narrow field parcels (so-called ‘*Hufen*’,^46^). A harbour site (area E, Fig. 2a) relates to remains of a major sea dike^23^, protecting the whole area against flooding. All four settlement areas thus belong to one large contiguous embanked and cultivated area, forming a so-called *polder*. Archaeological finds^30^ (see examples in Fig. 2b), radiocarbon ages^21,23,27^ and dendrochronology^21^ consistently date to the 12th to 14th cent. AD and thus place the settlement in the period of intense high-medieval land reclamation in North Frisia. Within the reconstructed polder and in the central part of the settlement area, magnetic gradiometry finally revealed the outline of a prominent former terp (Fig. 2a, labelled D) with a number of intriguing structures (Fig. 3a).

Despite an advanced state of erosion, our comprehensive reconstruction of natural conditions and man-made archaeological structures from the Südfall tidal flats (Fig. 2a) allows in combination with archaeological data for the first time a solid evaluation of the settlement area associated with Rungholt and its surroundings. Our results reveal a high settlement density with so far 64 terps, extensive cultivation measures reflected by a dense network of drainage ditches. This settlement pattern now connects directly to the well investigated large coastal protection works and harbour site south of Südfall^23^.

### Uncovering a church foundation hidden in the tidal flats

On this central terp (Fig. 3a), two magnetic anomalies stand out in particular: one rectangular (labelled I) and one semi-circular structure (labelled II). The amplitude plot (Fig. 3b) for an exemplary profile crossing both features I and II shows amplitudes that are at least one order of magnitude higher than the average background signal of the whole settlement area (cf. Fig. 2a). Frequency-domain electromagnetic induction (FD-EMI) prospection of the central terp area provides apparent conductivity maps (Fig. 3c) and corresponding apparent magnetic susceptibility maps (Fig. 3d), based on the calibrated In-Phase data, for three different depth ranges. Close to the surface (coil 1, down to approx. 0.5 m depth), shallow tidal creeks dominate the conductivity. For depths down to approx. 1 m (coil 2) and 1.8 m (coil 3), EMI results show an increased conductivity for the southern terp area, from which only feature I significantly deviates with lower values. Apparent magnetic susceptibility maps all show a high degree of detail. Several linear and rectangular, likely artificial structures appear on the northern part of the terp, but more striking is an area of increased susceptibility on the southern terp, framing relatively low susceptibility in features I and II. This area has a roughly E-W-orientated elongated shape, ca. 40 m long and 15 m wide, and shows a rectangular western and semi-circular eastern outline.

**Figure 4:**
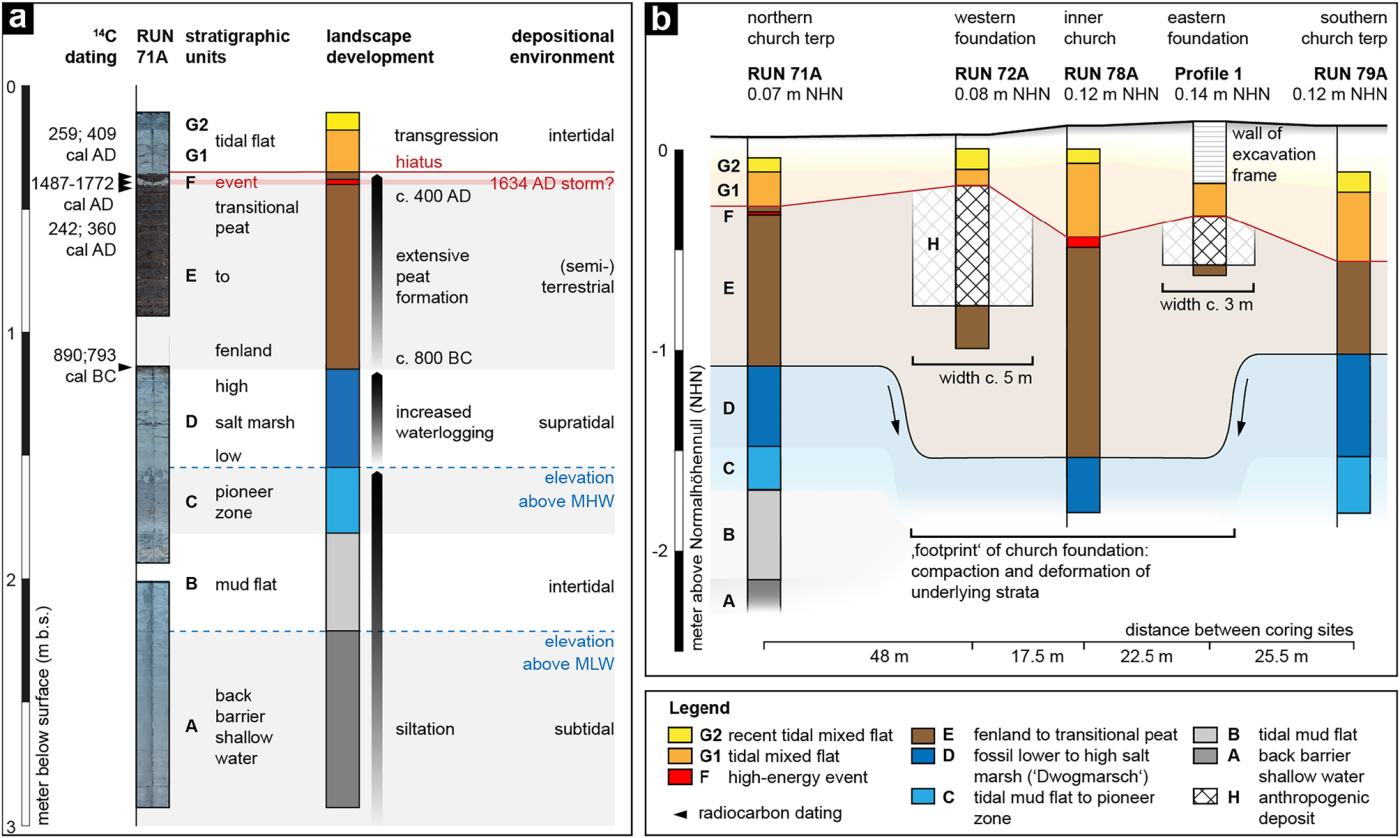
Stratigraphy, landscape development and human impact in the Rungholt area. (**a**) Core RUN 71A with its stratigraphic sequence shows a gradual landscape development from a back barrier shallow water environment to a high salt marsh (units A-D), without any evidence of human impact. Around 800 BC, waterlogging causes widespread peat formation (unit E) prevailing at least until 400 AD (cf.^22,27^) and forming a landscape hostile to settlers. A distinct hiatus and shift to tidal flat conditions (unit G) mark major land losses since medieval times. Sedimentary evidence of an early modern, likely the 1634 AD storm surge (unit F) was found in the upper peat while medieval deposits are missing. (**b**) At sites RUN 72A and Profile 1, artificially excavated depressions filled with shell debris and brick fragments (unit H) represent remains of a foundation. Compaction and deformation observed for soft underlying strata (units A-E) are interpreted as effects of superimposed load, i.e. a ‘footprint’ of the former structure on top (cf.^23^).

To calibrate geophysical prospection results, we retrieved four sediment cores from specific anomalies and dug one additional profile. Following sedimentary, geochemical and microfaunal palaeoenvironmental parameter analyses (see Supplementary Fig. S1, cf.^27^), we identified eight different stratigraphic units (A to H, Fig. 4, Supplementary Table S1), one of them of clearly anthropogenic origin (unit H). As expected, the terp’s stratigraphy is marked by a distinct hiatus (Fig. 4a), initiated by the 1362 AD storm and amplified by subsequent events and tidal processes: directly below (recent) tidal flat deposits (unit G) an erosively cut peat (unit E) dates from 800 BC to 400 AD (Table 1) while any remains of the medieval terp body are eroded. Typically for this region, the peat is underlain by a fossil salt marsh (unit D, cf.^27^) with high site-dependent variability in magnetic susceptibility (Supplementary Fig. S1 online), creating a characteristic patchy pattern in the magnetic map (Fig. 3a, e.g., southernmost part of the magnetic data example from area E). In contrast to the vertical sequence of natural autochthonous units (Fig. 4a), the rectangular and semi-circular shaped anomalies A and B are both clearly man-made structures (Fig. 4b). Considering their shape and distinctive composition of marine shell debris and gastropods as well as brick slags and fragments (unit H, Supplementary Table S1 online), the material was deliberately brought into the ground. Dug even below the former terp’s base into the peat subsoil, it strongly resembles foundation remains of a former building.

**Table 1:** Radiocarbon dating results.

Sample ID	Description	Depth	Depth	Lab. No.	$^{14}$C age	$\delta ^{13}$C	$2\sigma$ max; min
		(m b.s.)	(m a.s.l.)	(MAMS)	(yrs BP)	(ppm)	(cal BC/AD)
RUN 71A/3+ PR1	Plant remain (peat), undetermined	0.35	− 0.28	65629	1704 ± 17	− 31.4	259; 409 AD
RUN 71A/3+ PR1	Plant remain (peat), undetermined	0.40	− 0.33	65630	1755 ± 17	− 33.7	242; 360 BC
RUN 71A/3+ M	Articulated shell *(Cerastoderma edule)*	0.38	− 0.31	65631	944 ± 13	− 1.5	1487–1772 AD
RUN 71A/6+ PR	Plant remain (*Phragmites* sp.)	1.15	− 1.08	65632	2652 ± 19	− 26.8	890; 793 BC

b.s.—below ground surface; a.s.l.—above present sea level (NHN); Lab. No.—laboratory number; MAMS—Curt-Engelhorn-Center for Archaeometry, Mannheim; 2$\sigma$ max;min (cal BC/AD)—calibrated ages, 2$\sigma$ range; “;”—there are several possible age intervals due to multiple intersections with the calibration curve. Calibration based on Calib 8.2 software http://calib.org/calib/calib.html and calibration curves IntCal20^74^ and Marine20^75^. Reservoir correction (Delta R) for the German Wadden Sea, according to the marine database: Delta R -85 ± 17^76^.

Between structures I and II in the area of high susceptibility (Fig. 3d), we found the peat’s lower boundary to show a vertical offset of c. 0.4 m compared to the surroundings (Fig. 4b). Considering the soft subsurface strata (units A to E), this offset is interpreted as the effect of compaction and deformation by a superimposed load, i.e. a ‘footprint’ of the former (building) structure on top, also known for other nearby study sites nearby^23,47^.

**Figure 5:**
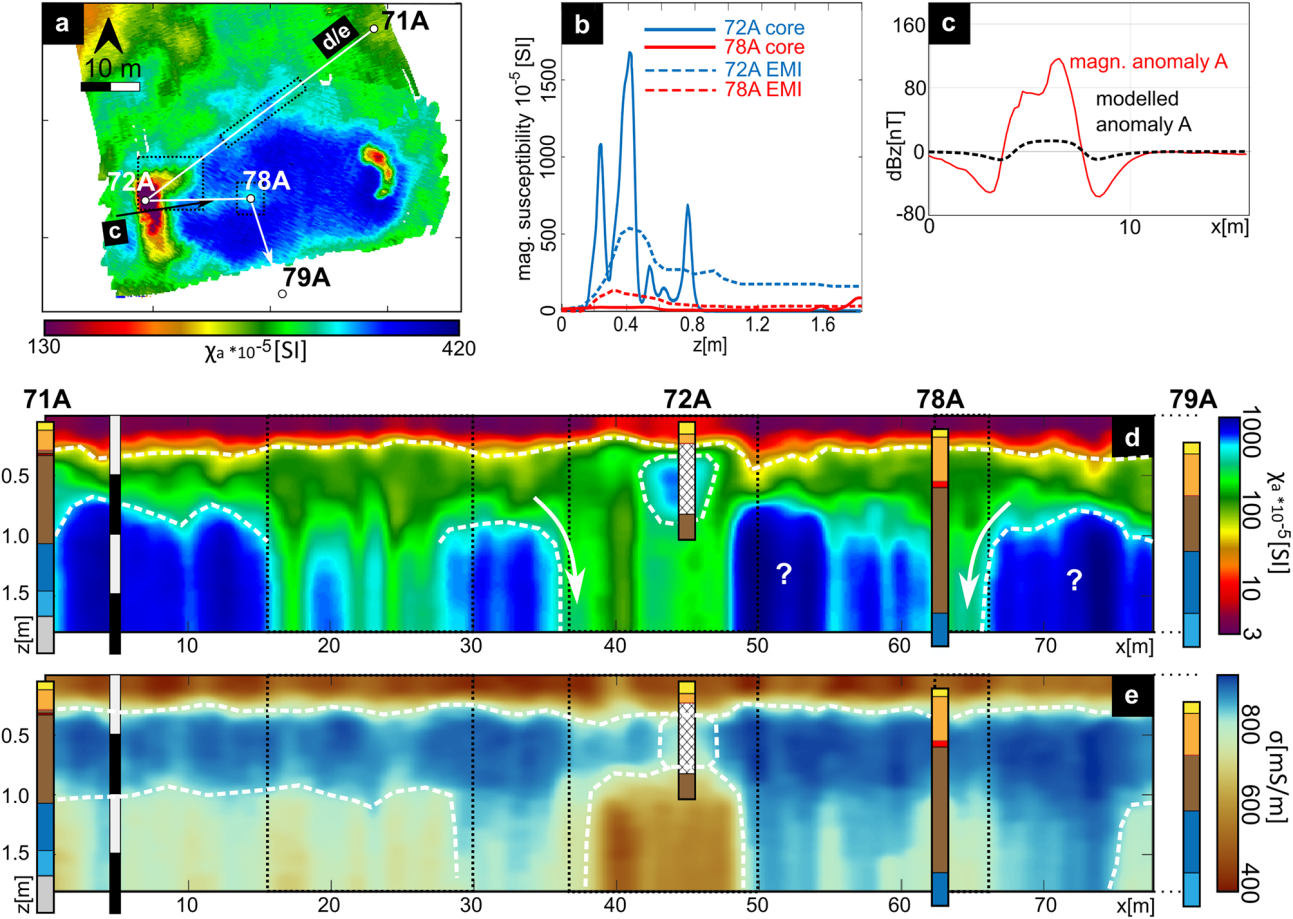
(**a**) A subset from the apparent magnetic susceptibility map of coil 1 from Fig. 3 including core positions (white dots) and selected example profiles for EMI inversion (labelled d/e) and magnetic forward calculation (labelled c). (**b**) Exemplary magnetic susceptibility values along cores 72A and 78A. Dashed lines display the EMI susceptibility inversion results. (**c**) Magnetic forward modelled anomaly A (dashed line) based on the measured core susceptibility values and the structure inverted from EMI in comparison to the measured magnetic anomaly (red line). (**d**) Result of the EMI profile magnetic susceptibility inversion. (**e**) Result of the EMI profile electric conductivity inversion result. In (**d**) and (**e**) all cores are added to the profile for interpretation purposes, as displayed in Fig. 4.

For continuous depth information on the observed anomalies and interpolation between coring sites, we combined EMI prospection results with core stratigraphies. We chose an exemplary (zig-zag) profile across the southern terp (Fig. 5a, white arrow), following cores 71A, 72A, 78A, and 79A and including structure I. Compared to magnetic susceptibility measured in all sediment cores, the EMI inversion at these points (Fig. 5b) clearly reconstructs the trend of the susceptibility distribution but also highlights the limited depth resolution of the method, probably due to the assumption of a 1D subsurface model and trade off effects in inversion. Nevertheless, the inversion basically reflects the general stratigraphic trend, giving insights into the spatial changes of the stratigraphic units along the inverted profile (Fig. 5d,e). Compared to the stratigraphic sequences at both ends of the transect, magnetic susceptibility inversion results show a three-layer case (Fig. 5d). The uppermost layer of lowest susceptibilities (purple to red) reflects recent tidal flat deposits (unit G). Below, moderate values (green colour) correspond to peat of unit E, while the lowermost highest values represent the fossil salt marsh (unit D). From c. 18 m to 30 m, the transect crosses several anomalies, well visible in the magnetic map. Here, the lower boundary of unit E seems to be disturbed. From c. 43 m to 48 m, a significant increase in susceptibility clearly corresponds to the presumed foundation remains (unit H, core 72A). Directly below, inversion results show a clear vertical displacement of the lower peat boundary from c. 38 m to 48 m and also 55 m to 65 m (core 78A), as already implied by the core transect (Fig. 4b). From c. 48 m to 55 m, strongly increased susceptibility values (Fig. 5a, labelled with question marks) remain enigmatic, but obviously frame the two features I and II. From 65 m to the end, outside the man-made structure, results again show the undisturbed three-layer case.

EMI electric conductivity inversion also shows a three-layer case (Fig. 5e). Results are influenced by tidal flat salt water dynamics and saturation as well as differences in the subsurface’s porosity. The uppermost layer of lowest conductivity reflects the sandy (recent) tidal flat deposits (unit G) that are easily drained and thus aerated during low tide. Below, highest electrical conductivities correspond to unit E peat, that shows both, a high water saturation and distinct enrichment of salt (Supplementary Fig. S1 online). Where the profile crosses structure I and coring site 72A, conductivity slightly decreases, likely due to the foundation’s coarse-grained character (unit H, cf. Supplementary Table S1 online). Below, from c. 38 m to 48 m (core 72A), we found a significant decrease of the peat’s otherwise high electrical conductivity. Considering the core transect, this effect is most likely also assigned to compaction and a decrease in pore volume caused by the superimposed load of the former structure on top. In between the two foundation structures, unit E is much thicker (cf. Fig. 4). Looking at the EMI conductivity maps (Fig. 3c), this creates an area of higher conductivity framing the two foundation remains.

From combined geophysical prospection and coring, we found the investigated terp to be fully eroded since medieval times. Only deeper man-made structures like the artificial layer of unit H (cf. Supplementary Table S1 online), reaching down into the peat (unit E) below the former terp, still remain. Based on the measured magnetic susceptibility and inverted cross section of structure I (Fig. 5b,d), we performed a magnetic gradiometry forward calculation^48^. From our results (Fig. 5c, dashed black line), the anomaly caused by the unit H layer (Fig. 3a) cannot be explained by the magnetic susceptibility contrast alone. In fact, the signal must also originate from an additional remanent magnetization of the observed structure. These foundation remains are also framed by an area of increased magnetic susceptibility of unknown origin and increased electric conductivity due to the building’s mechanical imprint on the sediments (here the high conductive peat), outlining a structure with curved eastern edge.

## Discussion

Prospecting drowned archaeological remains in the North Sea tidal flats is a challenging task. Only few noticeable areas of tidal flats with archaeological features were investigated from Denmark to Britain using different methodological approaches. Studies in Belgium involve, e.g., high resolution marine seismic prospection of a late Medieval settlement site^49^ or seismic measurements combined with terrestrial electromagnetic induction (EMI) and Cone Penetration Tests (CPT) at an intertidal test area^50^. Despite the different settings, these studies prove the potential of high resolution marine seismic methods and EMI to image the very shallow stratigraphies and archaeological objects of different scales. In the United Kingdom, the former medieval port and town of Dunwich were investigated by multibeam-, side-scan sonar-, and singlebeam sub-bottom seismic data^51^. In the Netherlands, fossil tidal flats, salt marshes and pre-Roman Iron Age to Medieval settlement remains are mostly situated behind modern dikes, and open for conventional archaeological prospection or excavations^3,18^. In Germany, different research approaches dealt with the Wadden Sea’s cultural heritage. In East Frisia, several surveys analysed aerial pictures, archaeological and geological data but also showed a first result of tidal flat magnetic gradiometry of a farmstead and tidal creek^52^. The authors of this study successfully imaged parts of the Rungholt settlement by combining magnetic gradiometry, marine reflection seismics and coring^23^ and also proved the potential of EMI measurements in North Frisia’s tidal flats^45^. The present study not only extends these approaches and for the first time creates a comprehensive picture of a drowned medieval settlement area, it also includes the first image of a large medieval building, preserved in a tidal flat environment. We found this settlement to be located in a coastal lowland, where pre-medieval, natural conditions were dominated by vast peatland (Fig. 4a). Starting around 800 BC, extensive peat formation lasted at least until the 4th cent. AD (Table 1), initially preventing settlement activities for many centuries^22,27^. While in some places the advancing North Sea already favoured the formation of new, fertile marshes in the first millennium AD^22^, peat formation continued locally until the early Middle Ages^25,53^. Despite the stratigraphic gap, our results for the Rungholt area clearly imply that medieval land reclamation was accompanied by an extensive extraction of peat for cultivation of underlying fossil salt marsh by Frisian settlers^27^. At some sites, terp construction obviously occurred on peat prior to or during cultivation (Fig. 2a, areas C and D). At other sites, we found evidence of medieval occupation directly on top of pre-800 BC marshland (Fig. 2a, area B^27,30^). Our reconstruction of land reclamation measures and the Rungholt area’s settlement pattern with rows of terps and elongated so-called ‘*Hufen*’ thus fits well to the typical medieval way of coastal marsh- and fenland colonization^46^. If we refer to the archaeological features of North Frisian high medieval dwellings described by e.g.^54^ and assume that the historical records on damage, victims, and survivors of the storm surge of 1634 in North Frisia^55^ are transferable to the 1362 situation, we can give a rough estimate of about 1000-1300 permanent inhabitants for the settlement reconstructed here so far (with 64 so far detected terps on 10 km^2^). Our results further confirm the major human impact and man-made transformation of a former natural coastal landscape. Cultivation measures certainly allowed a profitable agricultural use for a certain period^33^. Yet, on the long term they drastically raised its vulnerability against flooding^27^, culminating in the 1362 AD storm flood and land losses. Hence, the hiatus in North Frisia’s stratigraphy is in many ways also induced by human action.

**Figure 6:**
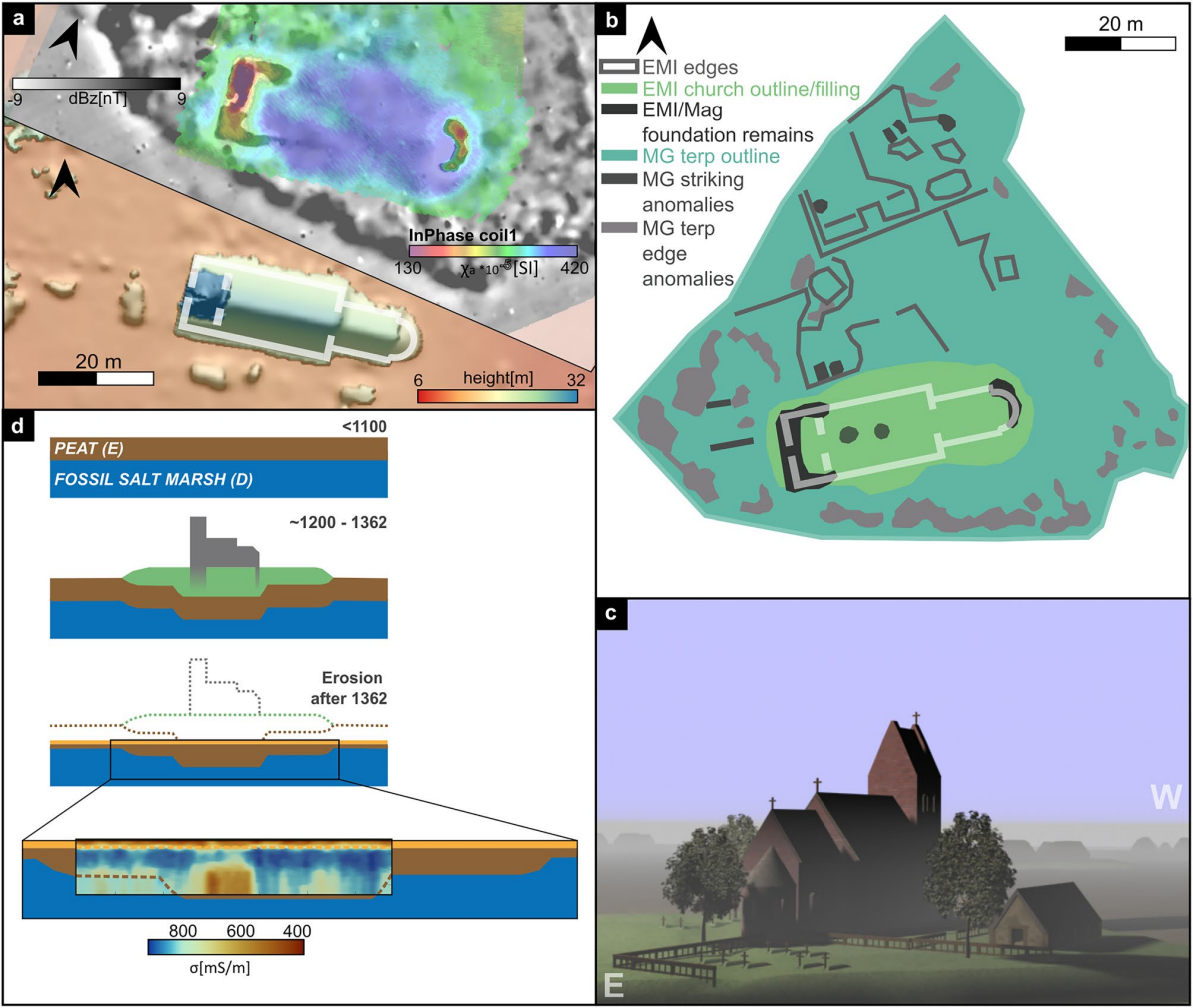
(**a**) Digital elevation model of the Breklum church (former medieval main church of the Nordergoesharde) (GeoBasis-DE/LVermGeo SH/CC BY 4.0) together with the geophysical results from the Rungholt area. The elevation model is superimposed by the ground plan of the Breklum church. (**b**) summary of all observed features in both magnetic gradiometry and EMI measurements. (**c**) Basic reconstruction of the church site based on comparable buildings from the same period. (**d**) model of the development of the church terp starting before Frisian colonization until today tidal flats together with the EMI inverted electrical conductivity profile.

The recent discovery of foundation remains on a prominent terp in the center of this medieval settlement area once again emphasise the site’s importance. The foundation’s characteristic outline revealed by geophysical prospection (Fig. 6a) implies that it is most likely a large, medieval church. To verify our hypothesis, we compared the shape and size of structures I and II with other medieval churches still preserved all across North Frisia. One excellent reference is, for example, the church at Breklum, that once served as main church for the medieval Nordergoesharde. Built around 1200 AD, this church still shows its original medieval layout and brickwork^56,57^. Comparing the newly discovered foundation structures I and II with a digital elevation model of the Breklum church (Fig. 6a) reveals striking similarities in size and shape: a large rectangular foundation to the west, a semi-circular apse to the east and a size of c. 15 m by 40 m. The Breklum church measures c. 13 m x 40 m with its western tower still being part of the original layout. The tower’s structural integration to the nave results in an unusual broad width. In general, the west towers are attached to the nave and significantly narrower. Breklum is so far the only known example of such a construction in North Frisia and its overall size stands out from the majority of North Frisian medieval churches. Among them, notable building lengths well above 30 m are only known for major churches, that were then also main churches of their respective harde (e.g., St. Salvador, Pellwormharde; St. Severin, Keitum/Sylt; St. Christian, Gardingharde^58,59^). This comparison clearly implies that the foundation remains discovered in the tidal flats must indeed be interpreted as the remnants of a Late Romanesque church with an integrated tower. The building must have been among North Frisia’s main churches and is most likely the one that provided a home and place of work for the clerical *collegium* documented in the sources^33,34,39^. Based on the distinct sacred architecture of Frisian churches of that time^34,57,58^, we created a first basic reconstruction of the church terp (Fig. 6c). Other obviously artificial features found in the geophysical datasets (Fig. 6b) indicate even more archaeological remains probably belonging to adjoining buildings and structures.

In between the two foundation structures I and II, peat (unit E) appears much thicker, as seen in the core transect (Fig. 4b) and the EMI inversion results (Fig. 6d). This most likely reflects the imprint of the former mechanical load of the building and terp on underlying stratigraphic units^23^, compacting and pushing down the peat that is thus preserved under the former terp and building. Looking at the EMI maps (Fig. 3c), this ‘footprint’ of pushed down peat frames the two foundation remains, leaving a rough outline of the building in the geophysical data.

Summarizing, recent research on the Rungholt settlement proves that an area strongly affected by intense erosion and sedimentary dynamics can still be an excellent archive for understanding human-environment interactions during the Middle Ages. The so far reconstructed three rows of terps, one harbour site, remnants of a major sea dike and finally a main church all imply a settlement of (supra-)regional importance that can well be associated with the settlement of Rungholt drowned in 1362 AD. But beyond this, the site is in any way a symbol and memorial for all coastal areas in the North Sea region, where intense man-environment interactions and human overexploitation of natural resources lead to their drowning by storm surges.

## Methods

This section explains the methodological approaches used for this work. Fieldwork was conducted in a mesotidal sand and mixed flats environment with terrain heights between 0.00 m and − 1.00 m NHN, creating a working time window of two to four hours per day around low tide. The sediment cover on top of the medieval landscape varies between 0.2 m (church site) and c. 2 m (wider surroundings). Acquiring the raw data for the church site and its direct surrounding (1.2 ha areal magnetic gradiometry, 0.4 ha EMI, 19.7 ha grid magnetic gradiometry, 9 corings, 1 test pit excavation) took 6 days of fieldwork. In total, the project dataset so far comprises approximately 235 ha of magnetic gradiometry (65 ha areal, 170 ha search grid), 30 ha reflection seismic grid, 2 ha of EMI measurements and c. 150 corings.

### Magnetic gradiometry

**Figure 7:**
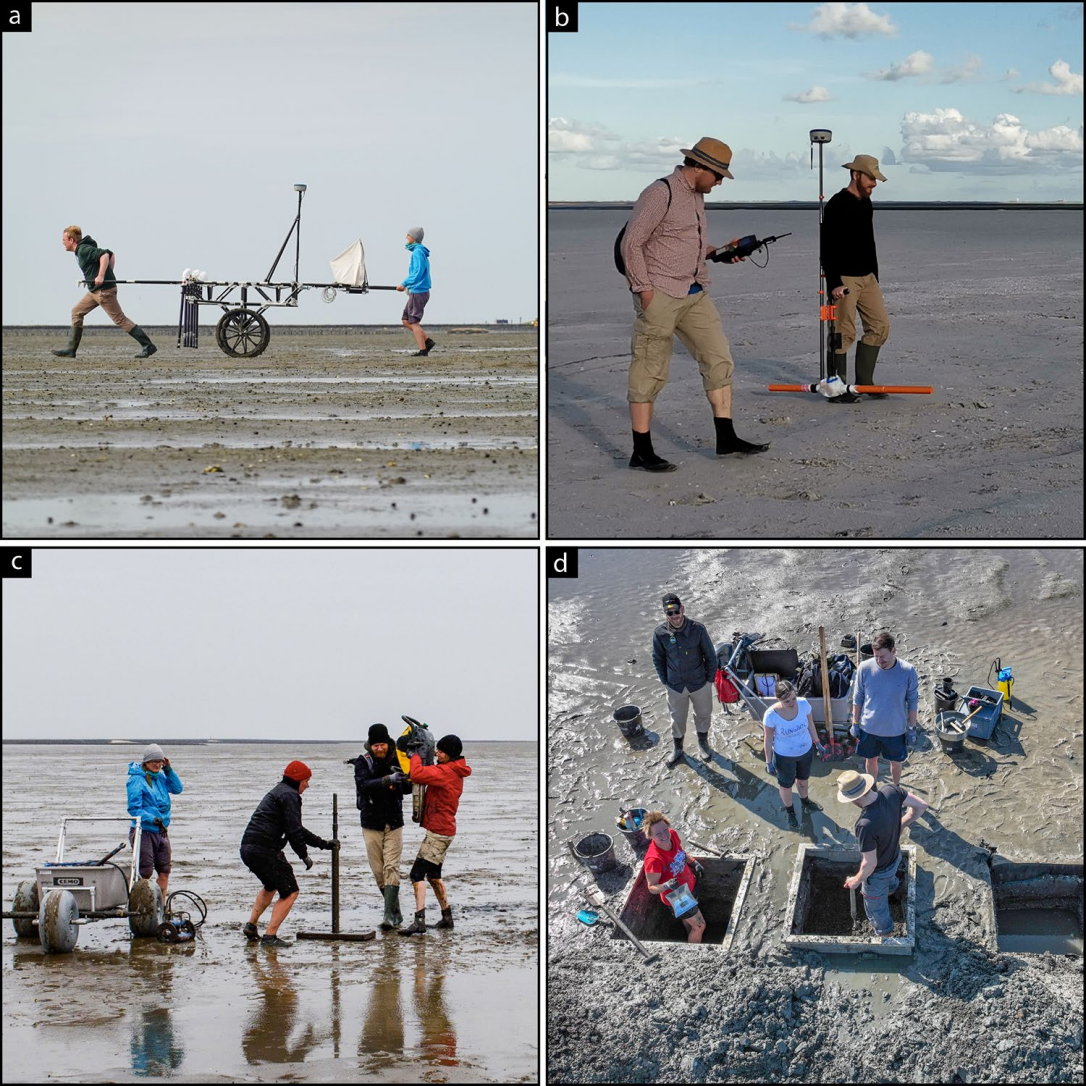
Methodological approach. (**a**) Magnetic gradiometry in the tidal flats during low tide (Photo: F. Schlütz, Kiel). (**b**) EMI prospection of sites selected from magnetic gradiometry results^45^. (**c**) Coring at specific sites selected from geophysical prospection results (Photo: J. Lemm, Berlin). (**d**) Test pit excavations to obtain soil profiles for characterization of sedimentary units and identification of potential archaeological structures. Informed consent was obtained from all subjects and/or their legal guardian(s) for publication of identifying information/images.

Magnetic gradiometry (Fig. 7a) measures the vertical difference of the vertical component of the magnetic field of the Earth. The difference is independent of regional fields and therefore mainly measures the local magnetic field caused by bodies in the shallow subsurface. A magnetic gradiometer survey was performed using an array of six Foerster fluxgate differential vertical gradiometers (vertical sensor distance of 0.65 m and horizontal sensor spacing of 0.5 m). Positioning was achieved by RTK (Real Time Kinematic) DGNSS (Stonex s9i device). Data processing follows a procedure developed by the authors of this study^23^.

### Electromagnetic induction (EMI)

Frequency-domain electromagnetic induction (EMI, Fig. 7b) devices are becoming more and more common in archaeological prospection (e.g.^60,61^). The EMI method uses electromagnetic waves generated in a transmitter and recorded in one or several receiver coils to create maps of electromagnetic properties of the subsurface for different sensing depths. The transmitter coil emits a ‘primary’ harmonic oscillating electromagnetic wave that induces eddy currents in the subsoil, depending on the electrical conductivity and the magnetic susceptibility of the soil. These eddy currents generate a ‘secondary’ field, which in superposition with the primary field is recorded at the receiver coils. Based on signal analysis, the EMI method provides apparent electrical conductivity of the subsurface and the so-called in-phase (IP) value, which is a function of the magnetic susceptibility (see e.g.,^62^) and conductivity in higher conductive regimes (e.g.,^63^). Different depth sensitivities can be achieved by using multiple receiver coils of different distances to the emitter coil, different coil orientations, varying height above ground, or different signal frequencies. The presented measurements were performed using a CMD (Multidepth Electromagnetic Conductivity Meter) Mini-Explorer by GF Instruments (Geophysical Equipment and Services). The device consists of one transmitter and three receiver coils. The planes of the coils were oriented horizontally (horizontal coplanar, HCP). The distances between the transmitter and receivers are 0.32 m, 0.71 m, and 1.18 m, respectively, resulting in three maps of apparent conductivity and IP values each. Further details on the method and the device can be found in^61^ or for tidal flat areas in^45^. The investigation area D (Fig. 2a) was covered by N-S-orientated tracks with an average spacing of about 0.5 m. An RTK-GNSS (Stonex S9i) was again used for positioning. Processing of the data included a) correcting RTK-GPS positions for each individual coil pair center offset, drift correction (after^64^), and gridding on a 0.2 m regular grid, followed by a 2D median image filter.

Sediment cores, positioned inside the EMI measurement area, were incorporated in a calibration process for the IP data. The cores were analyzed for magnetic susceptibility values using a Bartington MS3 instrument and MS2K sensor. At each coring site, EMI conductivity inversions were performed using the code of^65^, fitting theoretical data based on a 1D conductivity depth model to the three measured apparent conductivity values. The inverted conductivity as well as the measured susceptibilities were used to calculate theoretical IP data values. These values are then used to calibrate the IP data. This calibration is followed by a magnetic susceptibility inversion procedure. As the conductivity does not depend on the magnetic susceptibility, whereas in highly conductive material, the IP component is a function of both conductivity and magnetic susceptibility (e.g.^63^), we used the following inversion strategy. The data is first inverted for conductivity leaving the magnetic susceptibility at a constant half-space value. The result of this inversion is then used as a reference conductivity model in an inversion of the magnetic susceptibility.

### Coring and trenching

Based on geophysical prospection results, we selected nine different coring locations for the church site in order to identify prominent anomalies and for palaeolandscape reconstruction. Four cores are presented here as key sites directly connected to the building structure. Sediment cores were obtained using an engine-driven coring device (type Atlas Copco Cobra pro) and closed steel augers with plastic liners of 5 cm in diameter (Fig. 7c). All cores were opened, cleaned, photographed, described, and sampled in the laboratory. Descriptions of stratigraphic units followed standard procedures^66,67^ and comprised criteria like grain-size, sediment colour, carbonate content, macrofossil content, archaeological artefacts. Sedimentary logs were created using the GGU-STRATIG software (12.69, Civilserve GmbH, Steinfeld, Germany, https://www.ggu-software.com/geotechnik-software/feldauswertung/ggu-stratig-bohrprofil). Laboratory analyses comprised a project-specific set of sedimentary, geochemical and microfaunal paleoenvironmental parameters^27^ to assign each sedimentary unit to a specific facies: For all cores presented here, we obtained semi-quantitative element concentration using a pXRF instrument (Thermo Fisher Scientific Niton XL3t 900S GOLDD, calibration mode SOIL) at a 2 cm resolution, volumetric magnetic susceptibilities (Bartington MS2 instrument and MS2K sensor) and colour values (KonicaMinolta spectrophotometer CM-600d) at a 1 cm resolution. For sediment samples taken from the cores, we additionally determined grain size distributions, loss on ignition and electrical conductivity^68^. Determination and evaluation of ecological preferences of foraminifera and ostracoda were identified for core RUN 71A^69–72^. For detailed results see supplementary Fig. S1 and Table S1 online.

Natural conditions in the tidal flats (short time-windows during low tides, tidal creeks, accessibility) as well as sediment stability due to water saturation considerably limit the possibilities of test trenches to analyse soil profiles. We thus developed a novel, portable aluminium cofferdam of one by one meter size and 0.5 m height (Fig. 7d). It is pushed manually into the water-saturated permeable sandy topsoil and stabilizes the trench’s upper walls (Fig. 7d). This transfers the concept of common Test Pit Excavations (TPE) from dry land into the tidal flats and enables the targeted examination of selected geophysical anomalies, and stratigraphic profiles alongside extensive sampling. The approach was also tested and turned out suitable for documentation of archaeological features.

### Supplementary Information

Supplementary Information.

## Data Availability

The datasets generated during and/or analysed during the current study are not publicly available due to cultural heritage reasons but are available from the corresponding author on reasonable request.
