# Supplementary material for: The discovery of the church of Rungholt, a landmark for the drowned medieval landscapes of the Wadden Sea World Heritage
Source: Sci Rep. 2024 Jul 6;14:15576. doi: 10.1038/s41598-024-66245-0 (PMC11227575; doi:10.1038/s41598-024-66245-0)
Supplement: Supplementary file 1 — Supplementary Information. [file 41598_2024_66245_MOESM1_ESM.pdf]

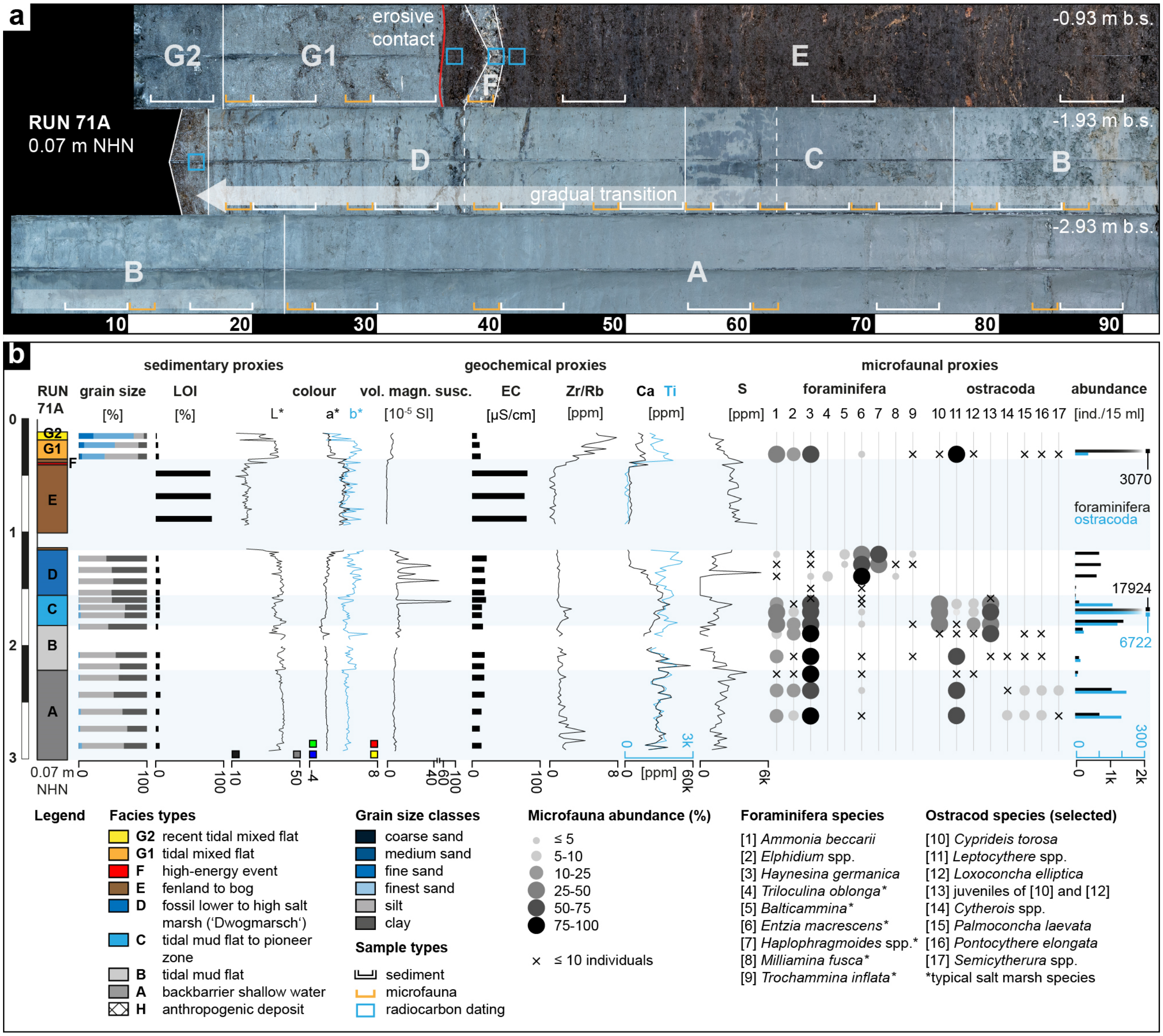

Supplementary Figure S 1. Stratigraphy (a) and palaeoenvironmental parameter analyses (PEP, b) for core RUN 71A.

Supplementary table S 1. Description of sedimentary units for the Rungholt church terp. All PEP (mean) values originate from RUN 71A analyses, as the core shows the most complete and undisturbed stratigraphy. Only unit H values originate from core RUN 72A. MS: magnetic susceptibility, EC: electrical conductivity, LOI: loss on ignition, Zr/Rb: Zirconium/Rubidium ration, ind./ml: individuals per milliliter

Unit	Sedimentary characteristics	Geochemical characteristics	Microfaunal characteristics	Cores RUN	Depositional environment	References
Unit A: back barrier shallow water	Soft, dark grey to grey (very) poorly sorted sandy mud with few plant remains and few *Peringia ulvae*. Mid to upper part quite homogeneous, lower part incorporates few thin sand layers (1 mm)	MS values are constantly low (avg. $5.92 \cdot 10^{-5}$ SI), while EC (avg. $5.960\mu m/cm$) slightly increases upward. LOI is at a moderate but constant level (avg. 9.15 %). Ti (avg. 1.546 ppm), Ca (avg. 30.451 ppm) and S (avg. 1.895 ppm) show a close correlation and slight upward increase. Zr/Rb (avg. 1.61) fluctuates in the lower and decreases in the upper part.	Hyaline species typical of tidal flat or estuarine environments[77] like *Haynesina germanica* accompanied by *Ammonia beccarii* and *Elphidium* spp. dominate the foraminiferal assemblage. Abundances vary throughout the unit (79 to 1072 ind./15 ml) but clearly decline towards the top. Species commonly associated with shallow water, lagoonal to open sea environments[72] like *Leptocythere* spp., accompanied by *Cytherois* spp., *Palmoconcha laevata*, *Pontocythere elongata* and *Semicytherura* spp. dominate the ostracod assemblage. Their abundance strongly declines at the top of the unit (225 to 8 ind./15 ml).	RUN 71A	sub- to intertidal	[22,27,72]
Unit B: tidal mud flat	Moderately firm, light grey to grey, poorly sorted (sandy) mud with few remains of roots. Very crumbly structure in upper part, homogeneous and rather soft in lower part. Weak mottling in lower part. Gradual lower and upper contact.	MS (avg. $8.06 \cdot 10^{-5}$ SI) and EC values (avg. $6.923\mu m/cm$) are still low but slightly increase upward. LOI remains at a moderate level (avg. 9.58 %). Following a decrease of both, Ti (avg. 1.687 ppm) compared to Ca (avg. 27.554 ppm), becomes slightly enriched upwards, while S (avg. 1.074 ppm) constantly decreases. Zr/Rb is rather low (avg. 1.21) with minor variations.	The microfaunal spectrum of this unit marks a gradual transition from unit A to C. *H. germanica*, *A. beccarii* and *Elphidium spp.* still dominate, accompanied by few individuals of the salt marsh associated *Entzia macrescens* and *Trochammina inflata*[78]. Foraminifera abundances clearly increase upward (110 to 1414 ind./15 ml). Ostracod abundances also increase upward (23 to 186 ind./15 ml). In the lower part, the assemblage still comprises *Leptocythere* spp. and few individuals of *P. laevata*, *P. elongata* and *Semicytherura* spp. Towards the top, these species are replaced by *Cyprideis torosa* and *Loxoconcha elliptica*, both typical for brackish lagoonal conditions[69,72].	RUN 71A	intertidal with lowest hydro-dynamic energy, likely (close to) former watershed	[79]

Unit	Sedimentary characteristics	Geochemical characteristics	Microfaunal characteristics	Cores RUN	Depositional environment	References
Unit C: tidal mud flat to pioneer zone	At the top firm, in the lower part rather soft brownish dark grey to dark grey (very) poorly sorted (sandy) mud with abundant in situ root remains and very fine shell debris. Distinct mottling in upper part, crumbly structure in lower part. Distinct but gradual lower and upper contact.	MS is slightly increased (avg. $15.69 \cdot 10^{-5}$ SI) with a local peak close to the upper boundary (max. $87.79 \cdot 10^{-5}$ SI). EC remains at mean level (avg. 5725 $\mu m/cm$), as does LOI (avg. 9.63 %). While Ti (avg. 1.748 ppm) shows a slight decrease, Ca (avg. 31.417 ppm) increases upward, as does S (avg. 1.584 ppm). Zr/Rb (avg. 1.53) increases mid-unit, corresponding to a slight shift in grain size.	In the lower part, both foraminifera (17924 ind./15 ml) and ostracoda (6722 ind./15 ml) reach a maximum abundance, that abruptly declines to the top of the unit (11 and 2 ind./15 ml, respectively). The unit's lower part is again marked by a dominance of *H. germanica*, accompanied by *A. beccarii* and *Elphidium spp.*, while only very few individuals of *H. germanica* and *E. macrescens* occur towards the top. The dominance of the ostracoda *C. torosa* and *L. elliptica*, accompanied by *Leptocythere* spp. continues, but like the foraminifera, they completely disappear to the top of the unit.	71A, 79A	intertidal with low hydrodynamic energy, decreasing flooding duration.	[22,79]
Unit D: fossil lower to high salt marsh ('*Dwogmarsch*')	In the lower part light grey, towards the top firm bluish grey and slightly marbled, poorly sorted mud with high avg. clay content (> 50 %). Lower part quite homogeneous, upper part with constantly increasing content of very well preserved *Phragmites* sp. remains, partly in thin layers (c. 5 mm). Gradual lower and distinct but gradual upper contact.	MS is significantly increased (avg. $13.49 \cdot 10^{-5}$ SI) with several distinct peaks (max. $44.54 \cdot 10^{-5}$ SI). EC (avg. 7648 $\mu m/cm$) shows a slight upward increase, as does LOI (avg. 10.66 %). While Ti (avg. 1.859 ppm) increases upwards, Ca (avg. 15.660 ppm) significantly decreases to a minimum. S (avg. 1.051 ppm) is low in the lower part, but strongly increases mid-unit upward. Zr/Rb (avg. 1,02) clearly declines.	Foraminifera are nearly absent at the unit's base but reach medium abundances (avg. 693 ind./15 ml) in the upper part. Here, species characteristic of (high) salt marsh environments like *Enzia macrescens* and *Haplophragmoides spp.*, accompanied by *Balticammina pseudomacrescens* and *Trochammina inflata* or *Triloculina oblonga* and *Miliammina fusca* dominate. Their distribution within the unit reflects the typical development from lower to high salt marsh conditions[78,80,81]. Ostracoda are completely absent.	71A, 78A, 79A	inter- to supratidal, semiterrestrial	[22,27,82]

Unit	Sedimentary characteristics	Geochemical characteristics	Microfaunal characteristics	Cores RUN	Depositional environment	References
Unit E: fenland (rich to poor fen)	Very compact and condensed, dark to reddish brown peat. Lower part with abundant macroscopic plant remains (esp. leaves and rhizomes of *Phragmites* sp.), upper part with remains of birch wood. Decomposition increases upward. Distinct, but gradual lower contact and erosive upper contact.	MS reaches minimum values (avg. $-0.46 \cdot 10^{-5}$ SI) while LOI is at a maximum (avg. 80,95 %). EC is exceptionally high (avg. 31.767 $\mu m/cm$, max. 42.500 $\mu m/cm$ in RUN 78A). Ti (avg. 113 ppm), Ca (avg. 4351 ppm) and Zr/Rb (avg. 0.46) are at a constant low level, while S values (avg. 3004 ppm) reach their maximum.	No samples taken.	71A, 72A, 78A, 79A, Profile 1	freshwater or brackish reed swamp, turned into salt peat by seawater intrusion	[10,83]
Unit F: high-energy event	Shell debris, valves and articulated shells (*Cerastoderma edule*) in grey sandy matrix. Layer intersects Unit E peat with sharp upper and lower contacts.	Due to the low thickness, geochemical parameters are not properly resolved for the unit.	No samples taken.	71A, 78A		e.g.[84]
Unit G, subunit G1: tidal mixed flat	Grey, (very) poorly sorted sandy mud to muddy sand with thin intercalations of OM, Fine shell debris and *P. ulvae* (1-5 mm). Coarsening upward and distinct contact towards overlying G2.	MS (avg. $4.05 \cdot 10^{-5}$ SI) is rather low, as are EC (avg. 4680 $\mu m/cm$) and LOI (avg. 5.11 %), both even decreasing upward. Ti (avg. 1551 ppm) is high, and Ca (avg. 14076 ppm) also increased, while S values (avg. 2076 ppm) decrease upward. Zr/Rb (avg. 4.34) increases with increasing grains size.	Typical tidal flat foraminifera occur in high abundance (3070 ind./15 ml), dominated by *H. germanica*, *A. beccarii*, *Elphidium* spp. (mostly *E. williamsonii*, *E. incertum*) and with few individuals of *E. macrescens*, *Lagena sp.* and *Ammoscalaria runiana*. Ostracod abundance is rather low (57 ind./15 ml) and as typical for recent tidal flats[81], *Leptocythere* spp. the dominant genus. Single individuals also occur of *C. torosa*, *L. elliptica*, *P. laevata*, *P. elongata* and *Semicytherura* spp.	71A, 72A, 78A, 79A, Profile 1	intertidal, mixed flat	[85][81]

Unit	Sedimentary characteristics	Geochemical characteristics	Microfaunal characteristics	Cores RUN	Depositional environment	References
Unit G, subunit G2: recent tidal mixed flat	Blackish grey, poorly sorted muddy sand with few fragments of *C. edule* and plant remains (mostly seaweed), strong smell of hydrogen sulphide (H_2S).	MS (avg. $3.64 \cdot 10^{-5}$ SI) remains at low level. EC (2770 $\mu m/cm$) and LOI (1.78 %) both reach lowest values of all units. Ti remains high (avg. 1324 ppm) and Ca increased (avg. 14.390 ppm). S (avg. 1169 ppm) declines to lowest values, while Zr/Rb (avg. 6.53) reaches a maximum.	No samples taken.	71A, 72A, 78A, 79A, (Profile 1)	intertidal, mixed flat	[81, 85]
Unit H: anthropogenic	Compact mixture of shell debris (mostly *C. edule*, but also intact individuals of the common periwinkle Littorina littorea), brick slag and brick fragments, brownish grey to rust-coloured, slightly layered. Sharp lower and upper contact.	MS values are significantly increased (avg. $488.83 \cdot 10^{-5}$ SI) and reach distinct maxima (up to $1913.33 \cdot 10^{-5}$ SI). EC (avg. 5656 $\mu m/cm$) and LOI (avg. 9,02 %) are at mean level, as is Ti (avg. 813 ppm).Ca reaches maximum values (avg. 41.725 ppm), while S (avg. 2310 ppm) and Zr/Rb (avg. 2.89) are slightly increased.	No samples taken.	RUN 72A, Profile 1		
